# Supplementary material for: Defective NADPH production in mitochondrial disease complex I causes inflammation and cell death
Source: Nat Commun. 2020 Jun 1;11:2714. doi: 10.1038/s41467-020-16423-1 (PMC7264245; doi:10.1038/s41467-020-16423-1)

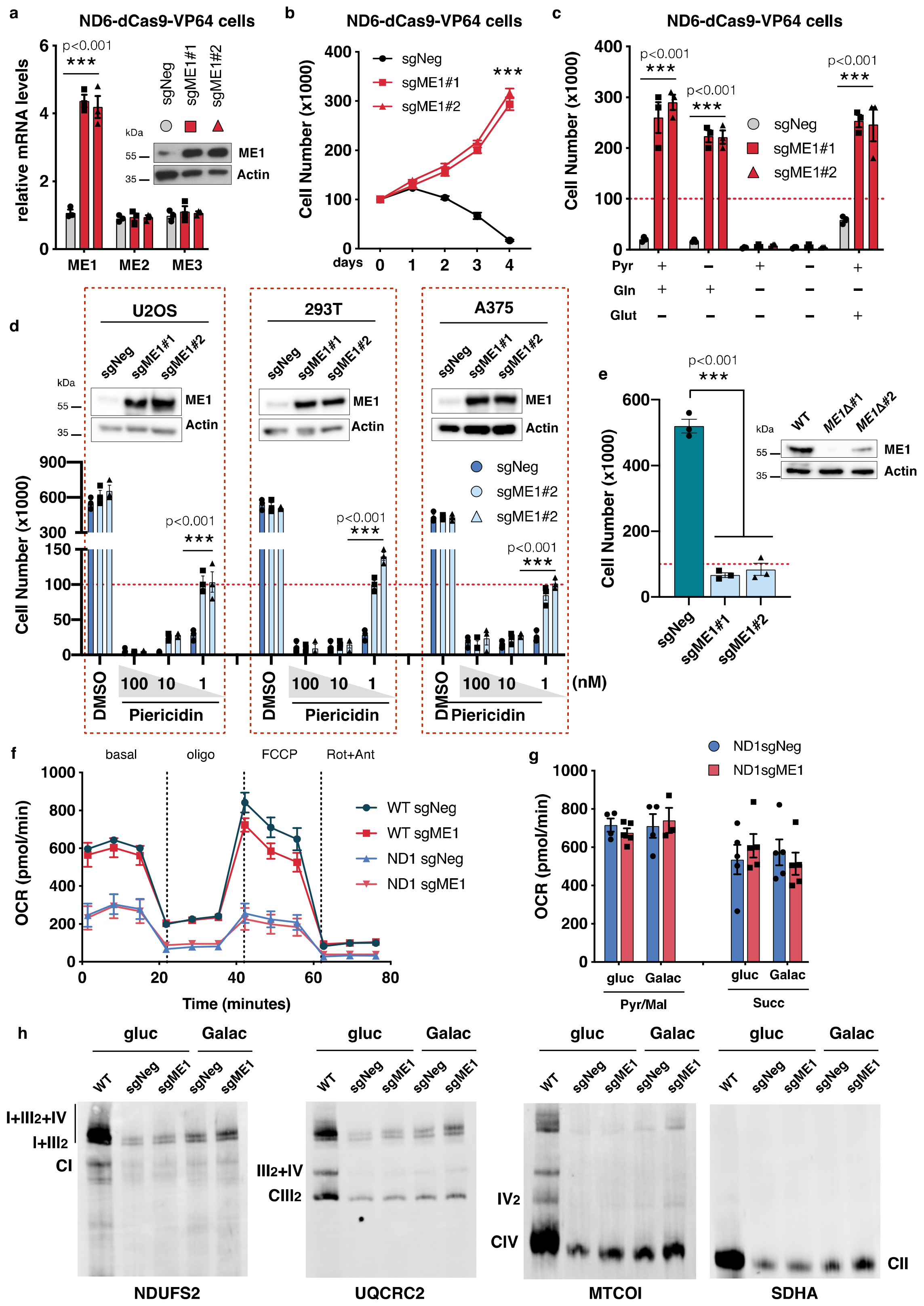
a
ND6-dCas9-VP64 cells
relative mRNA levels
p<0.001
***
sgNeg
sgME1#1
sgME1#2
kDa
55
35
ME1
Actin
ME1
ME2
ME3
b
ND6-dCas9-VP64 cells
Cell Number (x1000)
sgNeg
sgME1#1
sgME1#2
***
days
c
ND6-dCas9-VP64 cells
Cell Number (x1000)
p<0.001
***
sgNeg
sgME1#1
sgME1#2
Pyr
Gln
Glut
d
U2OS
293T
A375
sgNeg
sgME1#1
sgME1#2
kDa
ME1
Actin
Cell Number (x1000)
sgNeg
sgME1#2
sgME1#2
p<0.001
***
DMSO
100 10 1
(nM)
Piericidin
e
p<0.001
***
Cell Number (x1000)
WT
ME1Δ#1
ME1Δ#2
kDa
ME1
Actin
sgNeg
sgME1#1
sgME1#2
f
basal
oligo
FCCP
Rot+Ant
OCR (pmol/min)
WT sgNeg
WT sgME1
ND1 sgNeg
ND1 sgME1
Time (minutes)
g
ND1sgNeg
ND1sgME1
OCR (pmol/min)
gluc
Galac
gluc
Galac
Pyr/Mal
Succ
h
gluc
Galac
WT
sgNeg
sgME1
sgNeg
sgME1
I+III2+IV
I+III2
CI
III2+IV
CIII2
IV2
CIV
CII
NDUFS2
UQCRC2
MTCOI
SDHA

Supplementary Fig. 1 a, Specific mRNA and protein induction of ME1 using two different guides in ND6 mutant cells (n=3). **b**, Cells survival and proliferation curves in ME1 overexpressing ND6 mutant cells cultured in galactose media (n=3). **c**, Cell survival in ME1 overexpressing ND6 cells cultured under galactose media for 96 h in the presence of absence of glutamine (4mM), glutamate (4mM) or pyruvate (1mM) (n=3). **d**, ME1 overexpression rescue cell death in U2OS, 293T and A375 cells after pharmacological inhibition of Complex I using different doses of Piericidin (n=3). **e**, Genetic ablation of ME1 using CRISPR/Cas9 technology sensitize WT cells to galactose-induced cell death (n=3). ME1 expression does not alter respiration in **f**, intact cells or **g**, isolated mitochondria (n=5). **h**, BNGE analysis of digitonin-solubilized mitochondria showing that ME1 does not affect respiratory chain supercomplex levels in ND1 mutant cells. Immunoblots shown are representative of >3 independent experiments and all other experiments are represented as means ± SEM., n>3 biological replicates. Asterisks denote *p<0.05, **p<0.01 or ***p<0.001. Paired two-tailed Student's t-test in **a, b, e** and two-way ANOVA in **c, d**. gluc/g, glucose. Galac/G, galactose. Pyr, Pyruvate. Gln, Glutamine. Glut, Glutamate. Oligo, Oligomycin. Rot, Rotenone. Ant, Antimycin A. Red dashed lines indicate initial seeding density.

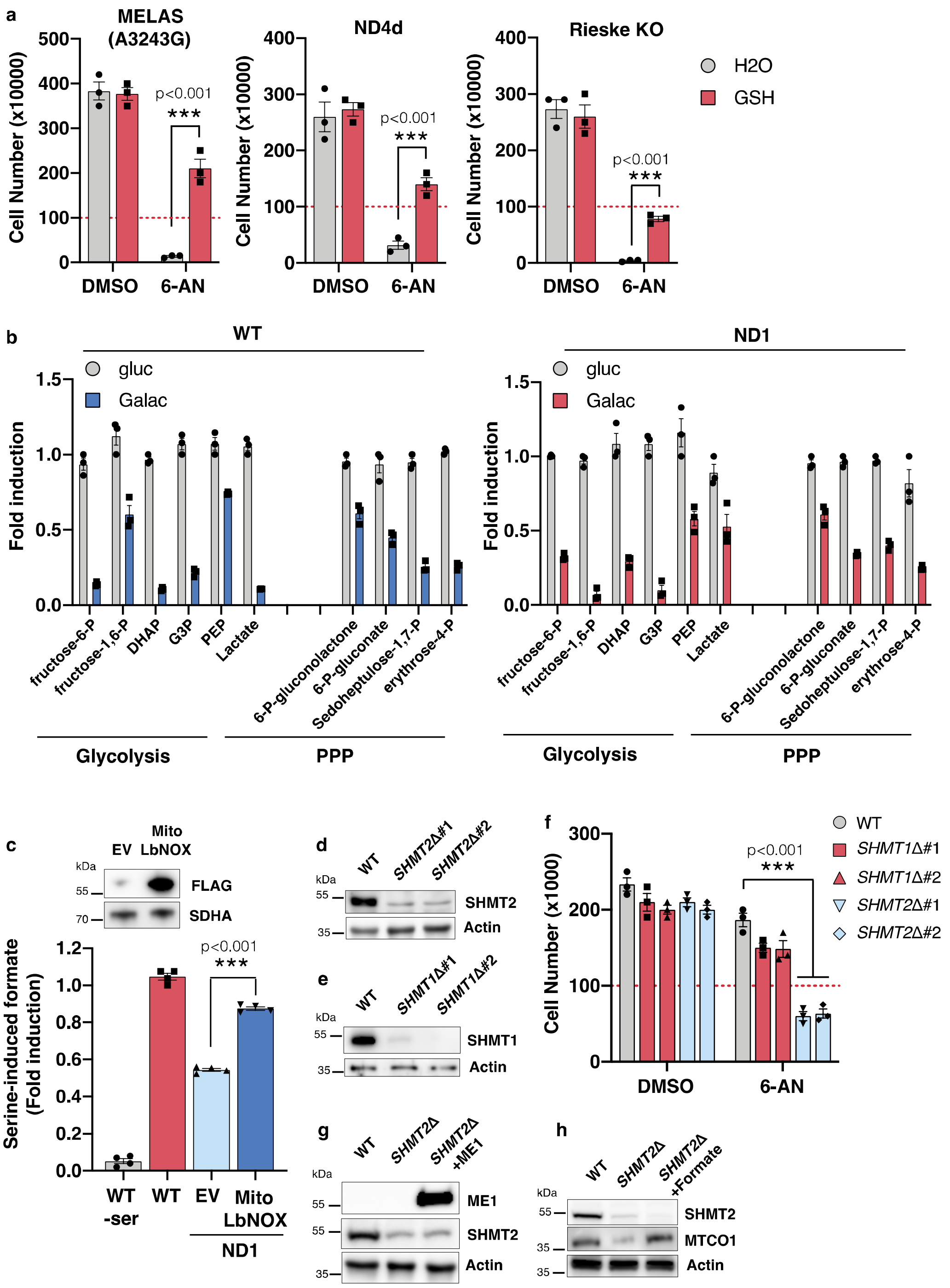
a
MELAS (A3243G)
ND4d
Rieske KO
Cell Number (x10000)
p<0.001
***
DMSO
6-AN
H2O
GSH
b
WT
ND1
gluc
Galac
Fold induction
fructose-6-P
fructose-1,6-P
DHAP
G3P
PEP
Lactate
6-P-gluconolactone
6-P-gluconate
Sedoheptulose-1,7-P
erythrose-4-P
Glycolysis
PPP
c
EV
Mito LbNOX
kDa
FLAG
SDHA
Serine-induced formate (Fold induction)
WT -ser
WT
EV
Mito LbNOX
ND1
d
WT
SHMT2Δ#1
SHMT2Δ#2
SHMT2
Actin
e
SHMT1Δ#1
SHMT1Δ#2
SHMT1
f
Cell Number (x1000)
SHMT1Δ#1
SHMT1Δ#2
SHMT2Δ#1
SHMT2Δ#2
g
SHMT2Δ
SHMT2Δ +ME1
ME1
h
SHMT2Δ +Formate
MTCO1

Supplementary Fig. 2 a, GSH (2mM) treatment rescued cell survival in mitochondrial mutant cells MELAS (A3243G-Leucine tRNA), ND4d (delA10227) and Rieske KO after 48 h inhibition of the PPP using 6-AN 100μM (n=3). **b,** Abundance of glycolytic and PPP intermediates in WT and ND1 cells cultured either in glucose or galactose for 24 h (n=3). **c,** Measurement of formate production from serine using isolated mitochondria from WT and ND1 cells expressing the NADH oxidase from *Lactobacillus brevis* (LbNOX) (n=4). Immunoblots showing reduction levels of **d,** SHMT2 or **e,** SHMT1 in corresponding CRISPR cells. **f,** Cell number of WT, SHMT1Δ and sgSHMT2Δ cells after inhibition of the PPP for 48 h (n=3). **g,** Immunoblot of SHMT2Δ cells after ectopic expression of ME1. **h,** Formate (1mM) restored impaired MTCO1 levels in SHMT2Δ cells. Immunoblots shown are representative of >3 independent experiments and all other experiments are represented as means ± SEM., n>3 biological replicates. Asterisks denote *p<0.05, **p<0.01 or ***p<0.001. One-way ANOVA in **a, c, f**. gluc/g, glucose. Galac/G, galactose. DHAP, Dihydroxyacetone phosphate. G3P, Glycerol 3-phosphate. PEP, Phosphoenolpyruvate. Red dashed lines indicate initial seeding density.

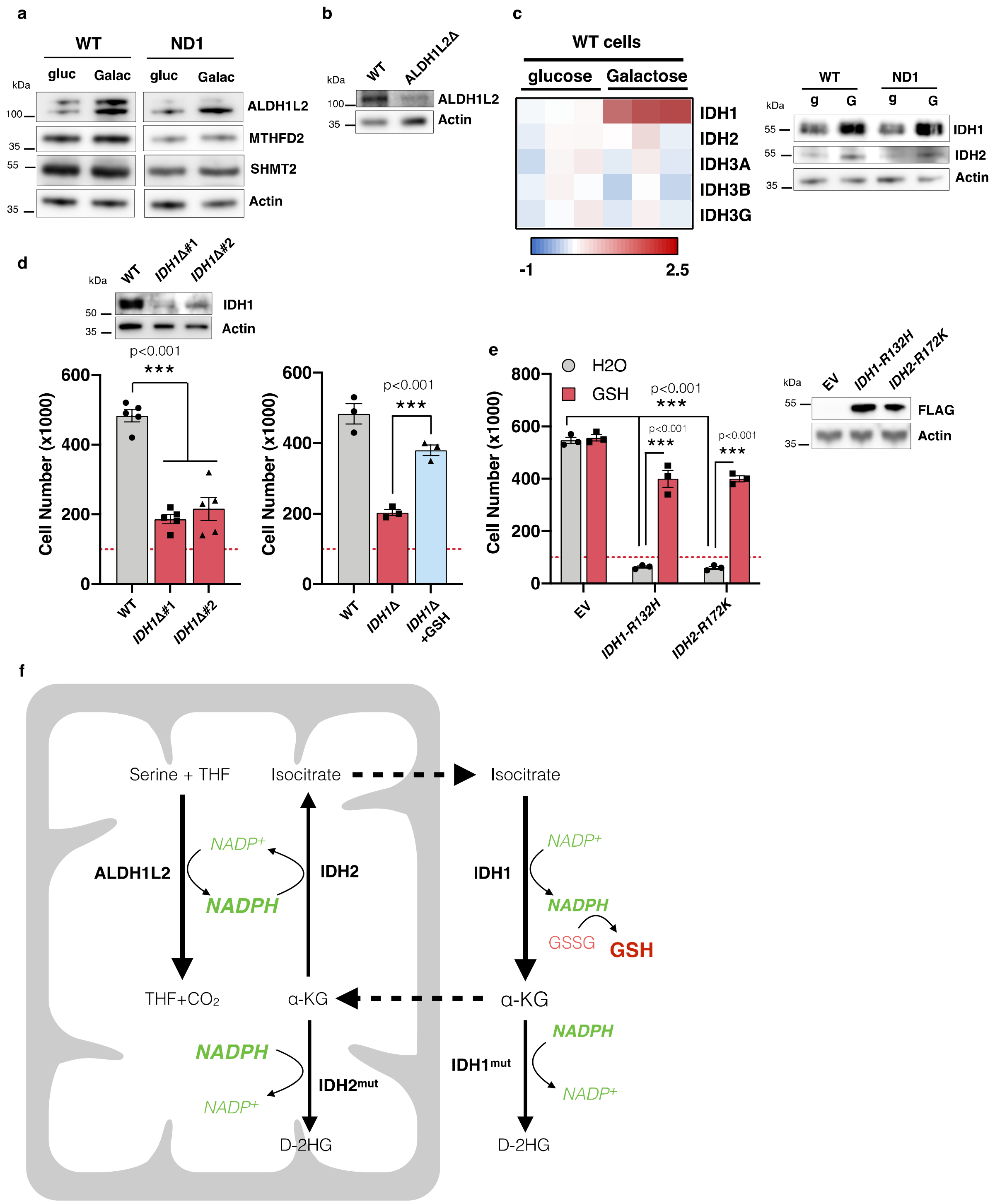
a
WT
ND1
gluc
Galac
gluc
Galac
kDa
100
35
55
35
ALDH1L2
MTHFD2
SHMT2
Actin
b
WT
ALDH1L2Δ
kDa
100
35
ALDH1L2
Actin
c
WT cells
glucose
Galactose
IDH1
IDH2
IDH3A
IDH3B
IDH3G
-1
2.5
WT
ND1
g
G
g
G
kDa
55
55
35
IDH1
IDH2
Actin
d
WT
IDH1Δ#1
IDH1Δ#2
kDa
50
35
IDH1
Actin
p<0.001
***
Cell Number (x1000)
600
400
200
0
WT
IDH1Δ#1
IDH1Δ#2
p<0.001
***
WT
IDH1Δ
IDH1Δ +GSH
e
H2O
GSH
p<0.001
***
800
600
400
200
0
EV
IDH1-R132H
IDH2-R172K
kDa
55
35
FLAG
Actin
f
Serine + THF
Isocitrate
Isocitrate
ALDH1L2
NADP+
NADPH
IDH2
IDH1
NADP+
NADPH
GSSG
GSH
THF+CO2
α-KG
α-KG
NADPH
NADP+
IDH2mut
IDH1mut
NADPH
NADP+
D-2HG
D-2HG

Supplementary Fig.3 a, Western blot of mitochondrial one carbon metabolism proteins. **b,** Levels of ALDH1L2 after genetic depletion using CRISPR/Cas9 technology. **c,** Proteomics heatmap (Left) and Western blot analysis (Right) showing increase protein content of IDH1, and to a lower extent IDH2. **d,** Decreased cell number in IDH1Δ cells culture in galactose for 96 h can be rescue by 2mM GSH supplementation (n=5). **e,** Ectopic expression of IDH1-R132H and IDH2-R172K mutant form sensitive WT cells to galactose-induced cell death that can be rescue by 2mM GSH treatment (n=3). **f,** Model proposing how IDH2 and IDH1 can work in synchrony to transfer ALDH1L2 generated NADPH from the mitochondria to the cytosol. IDH1 and IDH2 mutants create a futile cycle consuming WT-IDH1/2 produced NADPH. Immunoblots shown are representative of >3 independent experiments and all other experiments are represented as means ± SEM., n>3 biological replicates. Asterisks denote *p<0.05, **p<0.01 or ***p<0.001. One-way ANOVA in **d** and two-way ANOVA in **e**. gluc/g, glucose. Galac/G, galactose. EV, empty vector. THF, tetrahydrofolate. α-KG, alpha-ketoglutarate. D-2HG, D-2-Hydroxyglutarate. Red dashed lines indicate initial seeding density.

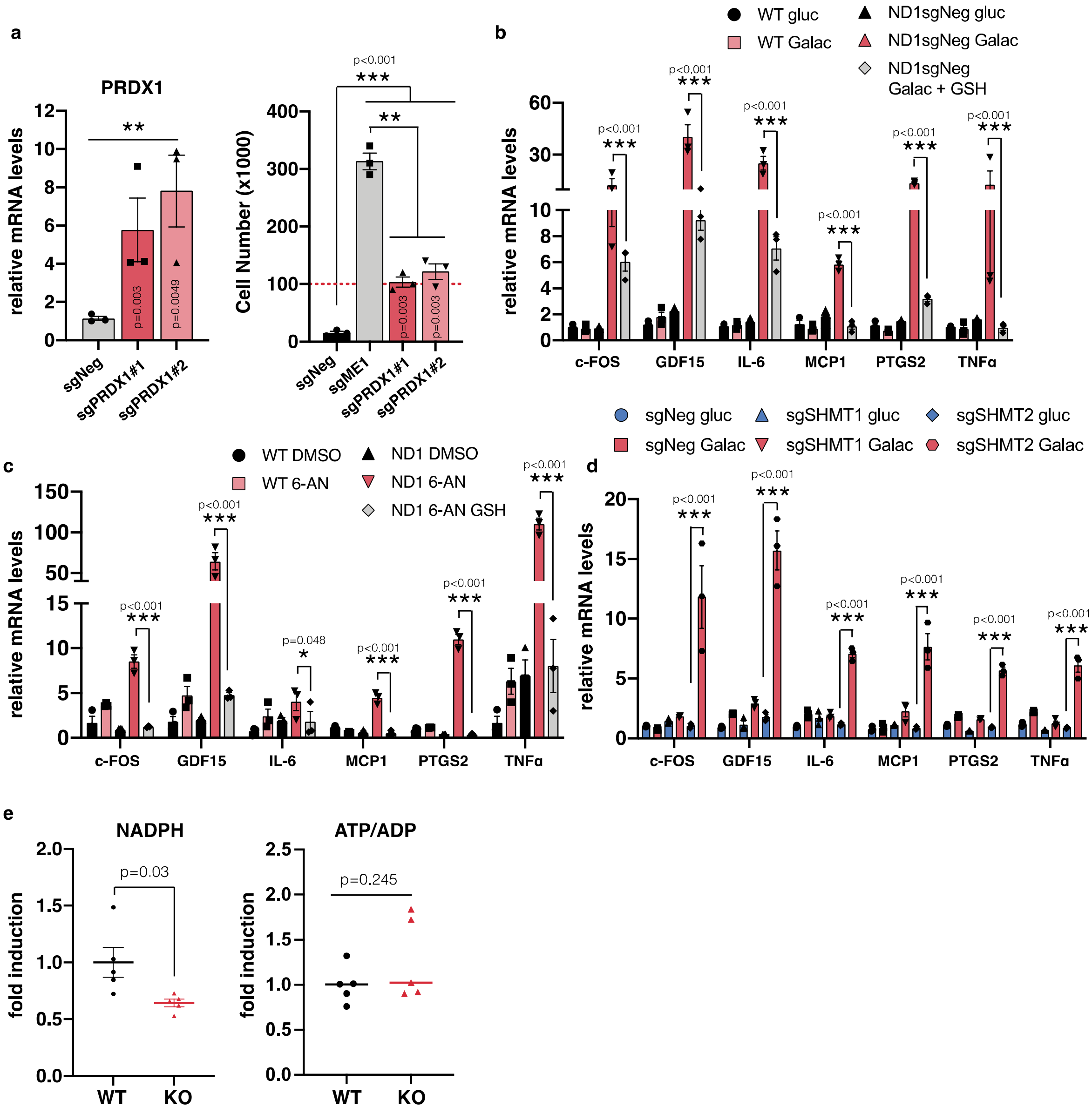

a
PRDX1
relative mRNA levels
**
p=0.003
p=0.0049
sgNeg
sgPRDX1#1
sgPRDX1#2
Cell Number (x1000)
p<0.001
***
**
p=0.003
p=0.003
sgNeg
sgME1
sgPRDX1#1
sgPRDX1#2
b
WT gluc
WT Galac
ND1sgNeg gluc
ND1sgNeg Galac
ND1sgNeg Galac + GSH
relative mRNA levels
c-FOS
GDF15
IL-6
MCP1
PTGS2
TNFα
c
WT DMSO
WT 6-AN
ND1 DMSO
ND1 6-AN
ND1 6-AN GSH
relative mRNA levels
p=0.048
c-FOS
GDF15
IL-6
MCP1
PTGS2
TNFα
d
sgNeg gluc
sgNeg Galac
sgSHMT1 gluc
sgSHMT1 Galac
sgSHMT2 gluc
sgSHMT2 Galac
relative mRNA levels
c-FOS
GDF15
IL-6
MCP1
PTGS2
TNFα
e
NADPH
fold induction
p=0.03
WT
KO
ATP/ADP
fold induction
p=0.245
WT
KO

Supplementary Fig.4 a, PRDX1 was overexpressed in ND1 mutant cells using two different guides (left) and promoted mild increased survival under galactose conditions (right) (n=3). **b,c,** Pro-inflammatory gene expression signature is induced in 48 h galactose-grown or PPP-inhibited ND1 mutant cells and rescue by 2mM GSH treatment (n=3). **d,** Inflammatory markers are induced in galactose-grown sgSHMT2 but not in sgSHMT1 cells (n=3). **e,** NADPH/NADP and ATP/ADP ratios are 0.0875+/-0.0259 and 2.8060+/-0.5539 (Average+/- Standard) respectively (n=5). Experiments are represented as means ± SEM, n>3 biological replicates. Asterisks denote *p<0.05, **p<0.01 or ***p<0.001. One-way ANOVA in **a** and two-way ANOVA in **b-d**. Red dashed lines indicate initial seeding density.

Figure 1d

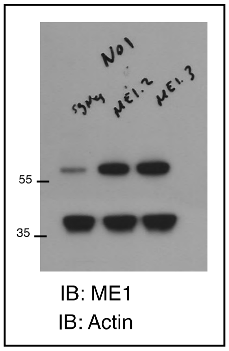

Figure 5a

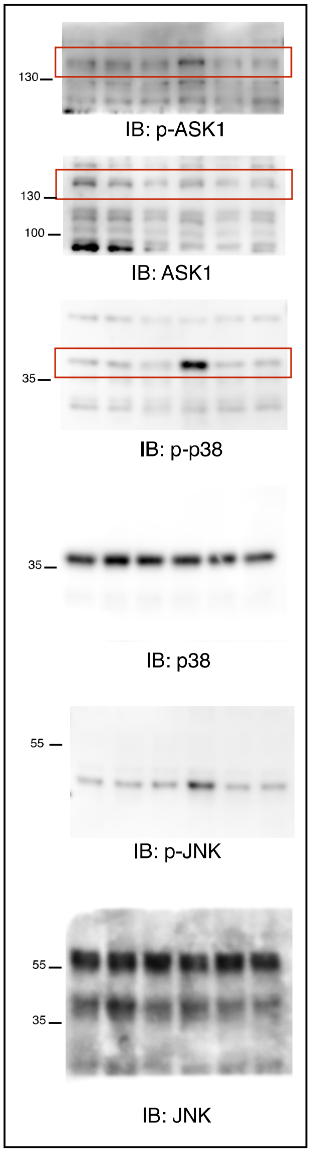

Figure 5b

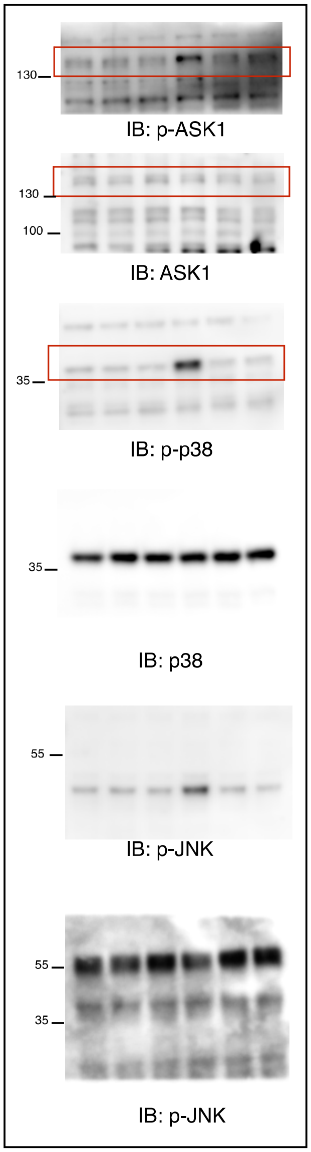

Figure 5f

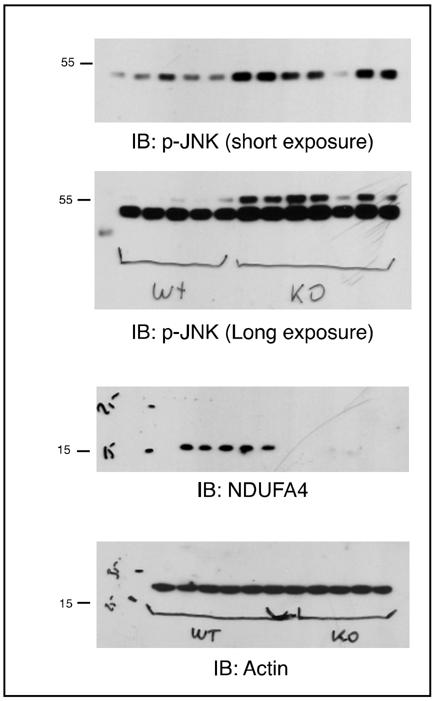

Supplementary Figure 1a

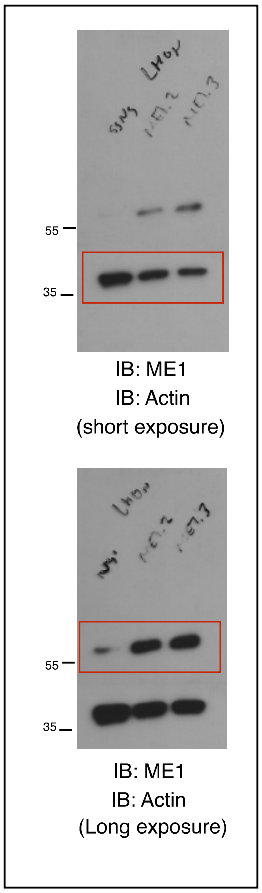

Supplementary Figure 1d

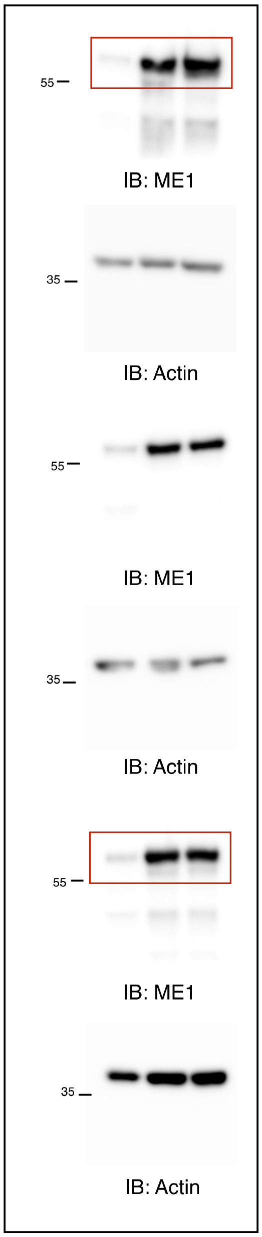

Supplementary Figure 1e

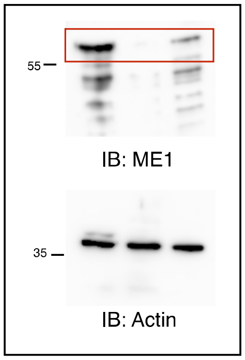

Supplementary Figure 1h

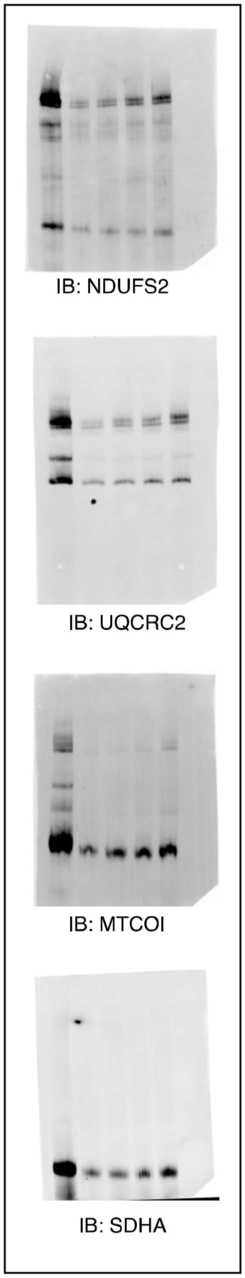

Supplementary Figure 2c

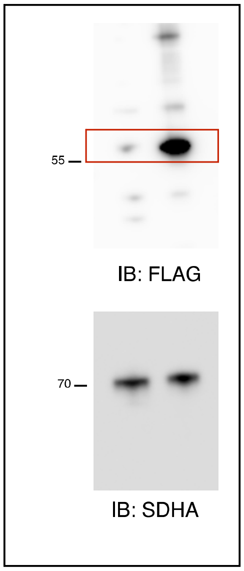

Supplementary Figure 2d

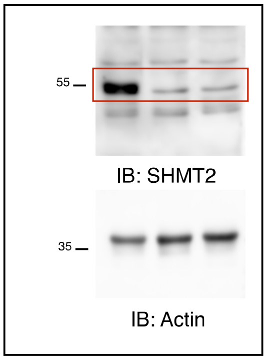

Supplementary Figure 2e

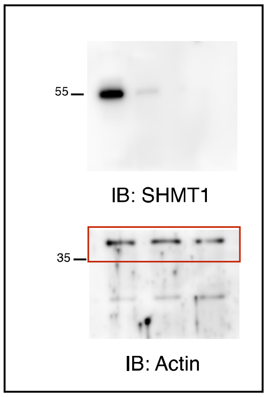

Supplementary Figure 2g

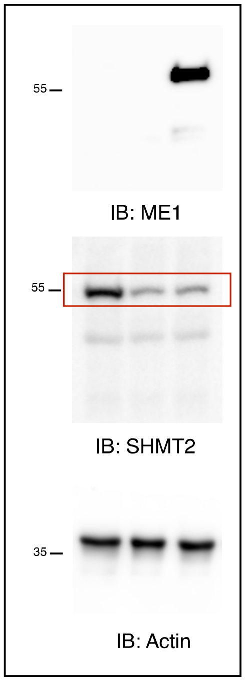

Supplementary Figure 2h

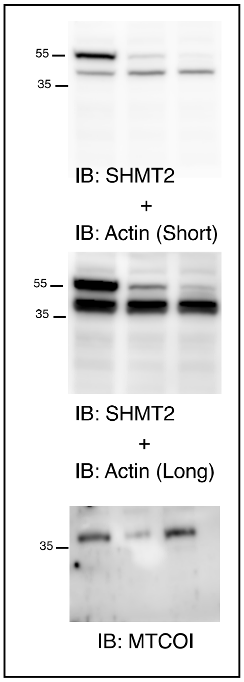

Supplementary
Figure 3a

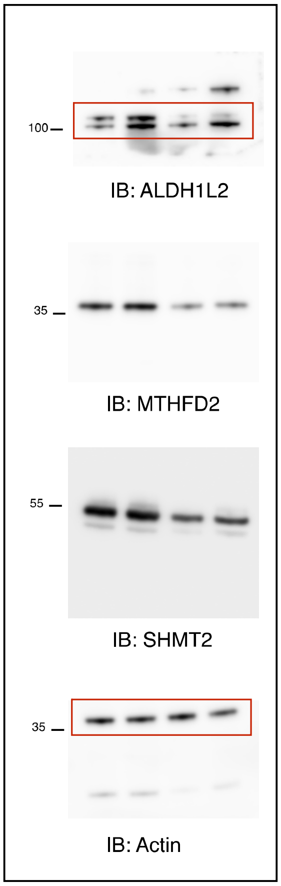

Supplementary
Figure 3b

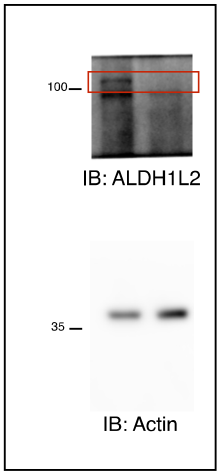

Supplementary
Figure 3c

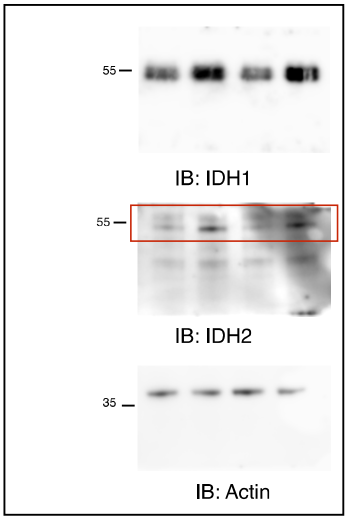

Supplementary
Figure 3d

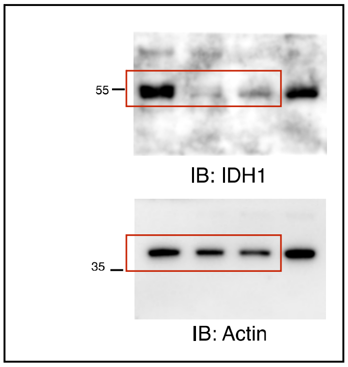

Supplementary
Figure 3e

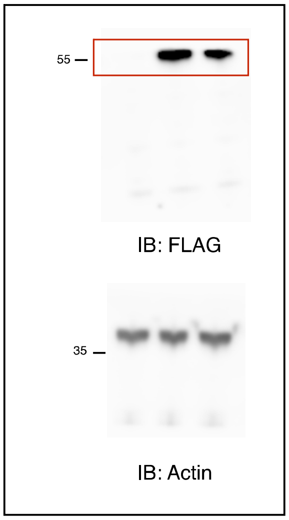

Supplement: Supplementary file 1 — Supplementary Information [file 41467_2020_16423_MOESM1_ESM.pdf]
