# Supplementary material for: Defective NADPH production in mitochondrial disease complex I causes inflammation and cell death
Source: Nat Commun. 2020 Jun 1;11:2714. doi: 10.1038/s41467-020-16423-1 (PMC7264245; doi:10.1038/s41467-020-16423-1)
Supplement: Supplementary file 3 — Description of Additional Supplementary Files [file 41467_2020_16423_MOESM3_ESM.pdf]

Description of Additional Supplementary Files

File Name: Supplementary Data 1.
Description: Supplementary Figures related to the manuscript as well as their corresponding legends and uncropped original scans.

File Name: Supplementary Data 2.
Description: Metabolomics analysis of Control and ND1 mutant cells.

File Name: Supplementary Data 3.
Description: Metabolomics analysis in brain samples of WT and Ndufs4 KO mice.

File Name: Supplementary Data 4.
Description: Proteomics analysis of Control U2OS cells cultured in glucose or Galactose conditions.
